# Vigorous Physical Activity Mitigates Susceptibility to Obesity Associated with Risk Genotypes of *FTO* and *MC4R*, and *SREBF1* Is Hypermethylated: A Cross-Sectional Pilot Study

**DOI:** 10.3390/epigenomes10020042

**Published:** 2026-06-21

**Authors:** Jenni Chambers, Mary Erazo Bastidas, Clare M. P. Roscoe, Corinna Chidley, Aaisha Makkar, Aparna Duggirala

**Affiliations:** 1 OMICS Facility, Biomedical and Clinical Sciences, School of Science, University of Derby, Derby DE22 1GB, UK; 2 School of Health, Sport, and Rehabilitation, University of Derby, Derby DE22 1GB, UK; c.roscoe@derby.ac.uk (C.M.P.R.);; 3 Department of Computing, School of Science and Engineering, University of Derby, Derby DE22 1GB, UK

**Keywords:** body fat percentage (BFP), body mass index (BMI), epigenetic, fat mass and obesity-associated gene (*FTO*), genotyping, melanocortin-4 receptor gene (*MC4R*), sterol regulatory element-binding transcription factor 1 gene (*SREBF1*), metabolism, methylation, obesity, physical activity (PA), pyrosequencing

## Abstract

**Aim**: The aim of this study was to correlate single-nucleotide polymorphisms (SNPs) in the *FTO* and *MC4R* genes with body composition (BC) in populations with various levels of physical activity, and to investigate associations of *SREBF1* methylation with the level of physical activity (PA) and BC. **Methods**: Fifty-six participants aged 18–65 years old with no underlying medical conditions were included in the study and were classified into sedentary/light PA (SLPA), moderate PA (MPA) and vigorous PA (VPA) groups using the International PA questionnaire (IPAQ). Anthropometric measures such as age, gender, body mass index (BMI) and body fat percentage (BFP) were recorded at the time of recruitment. Venous blood samples were collected during participant recruitment and DNA was extracted. Genotyping assays were performed for SNPs in *FTO* (rs9939609) and *MC4R* (rs17782313) using Taqman^®^ RT qPCR and TaqMan Genotyper software 1.7.1. Methylation analysis assay for CpG sites in the *SREBF1* gene was performed on 56 samples using PyroMark^®^ Q48 Autoprep (Qiagen, Venlo, The Netherlands). The results were statistically analysed to identify any associations between *FTO*/*MC4R* genotypes and the level of PA, and between *SREBF1* methylation status and the level of PA. This is the first study to investigate links between PA and quantitative methylation of *SREBF1*. **Results**: According to IPAQ guidance, the 56 participants were classified into SLPA *n* = 14, MPA *n* = 11 and VPA *n* = 31. The correlation analysis revealed that the *FTO* rs9939609 ‘A’ risk allele had a significant negative association with BFP in the VPA group (*p* = 0.0387); the *MC4R* rs17782313 ‘C’ risk allele had a significant positive association with BMI in the VPA group (*p* = 0.0256). In the *SREBF1* pyrosequencing analysis, higher levels of methylation were observed in the VPA group (*p* = 0.07). **Conclusions**: We concluded that SNPs associated with obesity identified in *FTO* rs9939609 and *MC4R* rs17782313 could help to predict the molecular effects of PA. A high frequency of *FTO* risk variants in the cohort was observed and the VPA group could help maintain a healthy BFP.

## 1. Introduction

Obesity is a significant global health challenge contributing to a range of diseases including cardiovascular disease (CVD), type II diabetes (T2D) and cancer [1]. Excess body fat is a hallmark of obesity attributed to an imbalance of energy intake and expenditure. Body mass index (BMI), calculated as kg/m^2^, is a commonly used measure in NICE classifications and clinical applications; a BMI over 25 kg/m^2^ is considered overweight and over 30 kg/m^2^ is considered obese [2]. Body fat percentage (BFP) is commonly estimated using bioelectrical impedance scales, calculated as total fat mass divided by total body mass. BFP is accepted as a useful body composition (BC) measure in addition to BMI as it distinguishes between tissue types; in trained individuals, increased muscle mass may contribute to a higher BMI, resulting in an inaccurate overweight or obese classification [3,4]. Physical activity (PA) is widely recognised as an important lifestyle intervention to combat weight gain and improve overall health, with UK government recommendations on weekly PA levels stated as at least 150 min moderate intensity, 75 min vigorous intensity, or a combination of both [5].

GWAS have played a useful role in understanding some of the molecular mechanisms involved with obesity. The development of obesity has been linked to the dysfunction of genes, such as the fat mass and obesity-associated gene (*FTO*), which was identified through the first genome-wide association study (GWAS) of its kind. *FTO* is involved in appetite regulation and energy homeostasis [6,7] and the regulation of several metabolic processes including the mTORC1 and AMPK pathways [8]. The melanocortin-4 receptor gene (*MC4R*) also has a strong association with BMI and obesity, regulating the leptin–melanocortin signalling pathway involved with satiety and energy homeostasis [9]. Single-nucleotide polymorphisms (SNPs) in the *FTO* and *MC4R* genotypes are recognised as a genetic risk factor for increased adiposity in phenotypes [10,11]. The *FTO* rs9939609 risk allele ‘A’ is associated with increased BMI and weight gain compared to the healthy wildtype ‘T’ allele [12,13], as is the *MC4R* rs17782313 risk allele ‘C’ in place of the healthy wildtype ‘T’ allele. The presence of one or two risk alleles in these gene SNPs has been found to contribute to the risk of unhealthy BMI or BFP through loss of function [14,15,16]. Regular PA is thought to help mitigate the obesity risk associated with *FTO* SNPs with a trend towards healthier body composition [17].

Epigenetic studies have further improved our understanding of obesity as a multifactorial disease, suggesting that environmental factors can modify a genetic predisposition to obesity [18]. PA can act as an epigenetic factor, influencing gene transcription and expression of genes associated with obesity through DNA methylation (DNAm) mechanisms [19,20,21]. PA initiates responses in a group of DNA methyltransferase enzymes including methylenetetrahydrofolate reductase, methionine synthase and methionine synthase reductase. These enzymes transfer methyl groups to cytosine–guanosine islands (CpG sites) in gene promoter regions and transcription start sites (TSS), which are involved in the regulation of transcription and gene expression [22]. An increase in methylation (hypermethylation) can inhibit or silence gene expression, while a reduction in methylation (hypomethylation) can allow increased gene expression through reduced gene regulation [23,24], and responses vary by tissue type [25]. Cholesterol biosynthesis is regulated by the sterol regulatory element-binding transcription factor 1 (*SREBF1*) gene. *SREBF1* is important for the regulation of lipid homeostasis and encodes a transcription factor involved in gene activation for fatty acid and cholesterol biosynthesis and metabolism [26,27]. The effect of PA on the epigenetic regulation of cholesterol biosynthesis genes remains unexplored. Hypomethylation of *SREBF1* has been identified as a mechanism for increased cholesterol biosynthesis in human adipocytes, resulting in greater accumulation of cholesterol [26]. Changes in the methylation of *SREBF1* associated with different levels of PA may help to improve our understanding of the role that PA plays in mitigating obesity risk. The literature evidence to date shows that *FTO/MC4R* and *SREBF1* are linked to BMI/ BFP and cholesterol metabolism, respectively, but their molecular link with the level of physical activity has not been established. Therefore, in this study, we hypothesise that the PA level can mitigate the risks associated with *FTO/MC4R* risk genotypes and alter the methylation signatures of *SREBF1* that are linked to cholesterol metabolism. The primary aim of the study is to genotype the SNPs in *FTO* and *MC4R* and correlate with the BMI, BFP and PA level. In addition, this study also aims to investigate the correlation of quantitative methylation of the *SREBF1* gene promoter with the BMI/BFP and level of physical activity.

## 2. Results

### 2.1. Cohort Characteristics

#### Fifty-Six Individuals Meeting the Inclusion Criteria Participated in the Study (Figure 1, Table 1)

Participants were between 18 and 65 years of age, males and females, non-smokers or -vapers, with no underlying health conditions or long-term prescription medications, and were not pregnant or lactating. Post hoc G*power analysis [28] indicated a study power of 0.6373738. Participants were classified by BMI according to NICE guidelines [2] (Table 2A). Participants were classified by BFP ranges according to the American College of Sports Medicine (ACSM) recommendations [29] with a distinction between male and female ranges (Table 2B). PA level groups were classified into sedentary/light PA (SLPA), moderate PA(MPA) and vigorous PA (VPA) based on International Physical Activity Questionnaire (IPAC) responses [30] (Figure 2).

**Figure 1:**
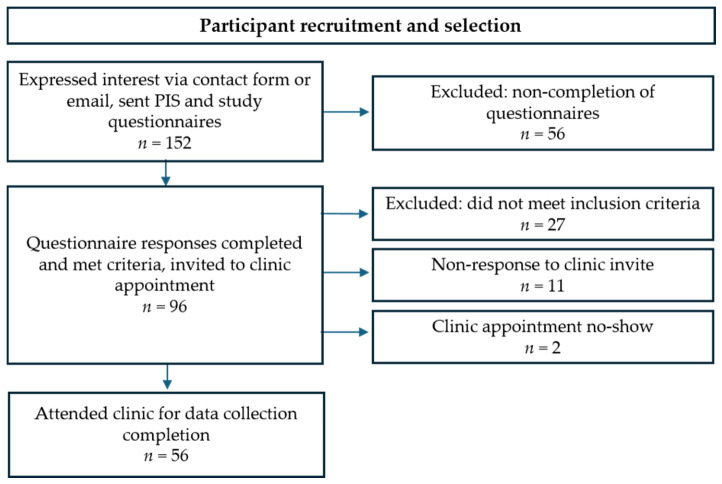
Participant recruitment and selection. In total, 152 individuals volunteered and 56 completed full participation in the study. *n* = 27 excluded for not meeting the criteria. Other participants were withdrawn due to non-completion of questionnaires (*n* = 56) or non-response to clinic invitations (*n* = 11).

**Table 1:** Participant characteristics. Results shown overall and by PA level group for anthropomorphic measures, *FTO* rs9939609 and *MC4R* rs17882313 genotypes, and *SREBF1* methylation M-values. Ranges shown with (mean) and ±standard deviation. Genotypes shown as *n* and percentage for total population and by PA group.

Characteristics	All	SLPA	MPA	VPA
Participants *n*	56	14	11	31
Female (%)	57.14	92.86	36.37	48.39
Age (years)	18–60 (32.38) ± 12.51	18–56 (33.57) ± 11.97	18–49 (30.18) ± 11.13	19–60 (32.61) ± 12.90
Height (cm)	155–194 (171.97) ± 9.59	157.5–188.5 (166.46) ± 8.01	163–194 (175.32) ± 8.57	155–187 (173.27) ± 9.44
Weight (kg)	48.4–105.2 (75.87) ± 13.65	53.8–105.2 (77.92) ± 12.11	57.5–102.4 (78.59) ± 13.03	48.4–77.5 (73.98) ± 14.01
BMI (kg/m^2^)	18.5–36.8 (25.67) ± 4.32	18.5–36.8 (28.26) ± 4.88	21.3–31.5 (25.47) ± 3.08	20.1–36.6 (24.58) ± 3.85
BFP (%)	7.5–44.4 (24.85) ± 9.67	12.3–41.7 (33.48) ± 7.74	9–33.5 (20.69) ± 7.09	7.5–44.4 (22.42) ± 8.69
*FTO* rs9939609 *n* (%)				
TT	13 (23.63%)	5 (35.71%)	3 (27.27%)	5 (16.67%)
AT	28 (50.90%)	5 (35.71%)	5 (45.46%)	18 (60.00%)
AA	14 (25.45%)	4 (28.58%)	3 (27.27%)	7 (23.33%)
*MC4R* rs17782313 *n* (%)				
TT	31 (55.35%)	8 (57.14%)	3 (27.27%)	20 (64.52%)
CT	23 (41.07%)	6 (42.86%)	8 (72.73%)	9 (29.03%)
CC	2 (3.57%)	0	0	2 (6.45%)
*SREBF1* CpG M-value (mean)	−2.70 to −0.92 (−1.95) ± 0.40	−2.70 to −1.45 (−1.98) ± 0.44	−2.58 to −1.64 (−2.19) ± 0.41	−2.45 to −0.92 (−1.85) ± 0.40

**Figure 2:**
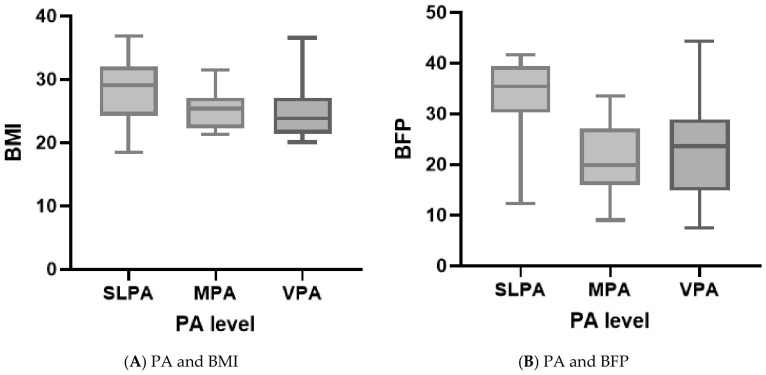
Cohort physical activity levels and body composition classification. Whisker box plots indicate distribution of BMI (**A**) and BFP (**B**) by PA level.

**Table 2:** (**A**) Participant classification of BMI by PA group. BMI was measured using a stadiometer and bioelectrical impedance scales and calculated as mass/height kg/m^2^. Grouping was based on NICE BMI guidelines for weight classification [2]. *N* shown as values and (%) of cohort. (**B**) Participant classification of BFP by PA group. BFP was estimated using bioelectrical impedance scales. Classification was based on American College of Sports Medicine (ACSM) BFP male and female ranges [29]. *N* shown as values and (% of cohort).

(**A**)				
**BMI Group**	**SLPA** ***n* = 14**	**MPA** ***n* = 11**	**VPA** ***n* = 31**	**Total** ***n* = 56**
Healthy	4 (7.14)	5 (8.92)	20 (35.71)	29 (51.78)
Overweight	4 (7.14)	5 (8.92)	8 (14.28)	17 (30.35)
Obese	6 (10.71)	1 (1.78)	3 (5.35)	10 (17.85)
(**B**)				
**BFP Group**	**SLPA** ***n* = 14**	**MPA** ***n* = 11**	**VPA** ***n* = 31**	**Total** ***n* = 56**
Athlete	1 (1.78)	2 (3.57)	8 (14.28)	11 (19.64)
Fitness	1 (1.78)	4 (7.14)	5 (8.92)	10 (17.85)
Average	3 (5.35)	3 (5.35)	13 (23.21)	19 (33.92)
Obesity	9 (16.07)	2 (3.57)	5 (8.92)	16 (28.57)

Data sets were checked for normal Gaussian distribution using the Shapiro–Wilk test. BMI and BFP data sets were analysed by PA level. The VPA group did not meet the threshold for normal distribution of BMI (*p* = 0.0090) and the SLPA group did not meet the threshold for normal distribution of BFP (*p* = 0.0068). The Kruskal–Wallis test indicated significant variation in the means for BMI (*p* = 0.0358) and BFP (*p* = 0.0004).

### 2.2. Genotyping

#### 2.2.1. SNP Frequencies

Allelic discrimination plots were generated by StepOneTM Software v2.2.2 and interpreted by TaqMan^®^ Genotyper Software (version 1.7.1). Frequencies of allele variants for each gene SNP are shown in Table 1. A QC fail for the genotyping assay excluded one sample from results for *FTO* rs9939609. The highest-frequency allele for *FTO* rs9939609 was the AT risk variant (*n* = 28); *MC4R* rs17782313 had the highest proportion of the healthy variant TT (*n* = 31).

The genotypic frequencies for *FTO* rs9939609 were found to be in Hardy–Weinberg equilibrium, with an A allele frequency of 0.509 and T allele frequency of 0.49. Genotypic frequencies for *MC4R* were in Hardy–Weinberg equilibrium with a C allele frequency of 0.241 and T allele frequency of 0.7589.

#### 2.2.2. Statistical Analysis

Odds ratios were calculated for risk variant presence or absence, and between healthy and unhealthy classifications of BC. A result of 1 was considered to have no association, while >1 was associated with a risk of increased BMI or BFP with the presence of a variant, and <1 indicated low or no associated risk of increased BMI or BFP with the presence of a variant. Strong associations were identified with *FTO* rs9939609 in the SLPA BMI overweight/obese group (2.333), and in the SLPA (2.000) and MPA (2.000) BFP average/obesity groups (Table 3A). Strong associations were identified with *MC4R* in all PA levels for BMI overweight/obese groups, particularly in the SLPA group (SLPA 3.000, MPA 1.762, VPA 1.944), and with VPA in the BFP average/obesity group (2.667) (Table 3B).

Correlation analysis was completed for *FTO* and *MC4R* risk alleles. Comparisons for each SNP were made between PA groups and BMI, and between PA groups and BFP. The effect of risk allele A in *FTO* rs9939309 was significantly negatively associated with the VPA level for BFP, *p* = 0.0387 (Table 4A). The effect of risk allele C in *MC4R* rs17782313 was significantly positively associated with the VPA level for BMI, *p* = 0.0256 (Table 4B).

Multiple logistical regression analysis was completed to check for confounding factors including age and sex and no significant differences were found.

### 2.3. Pyrosequencing

**Figure 3:**
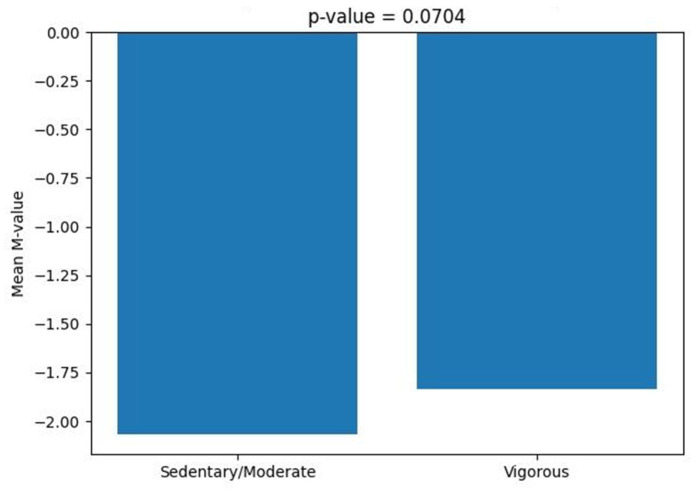
Comparison of M-values between SLPA/MPA vs. VPA groups shows that methylation levels in the promoter region of *SREBF1* are not significant.

**Figure 4:**
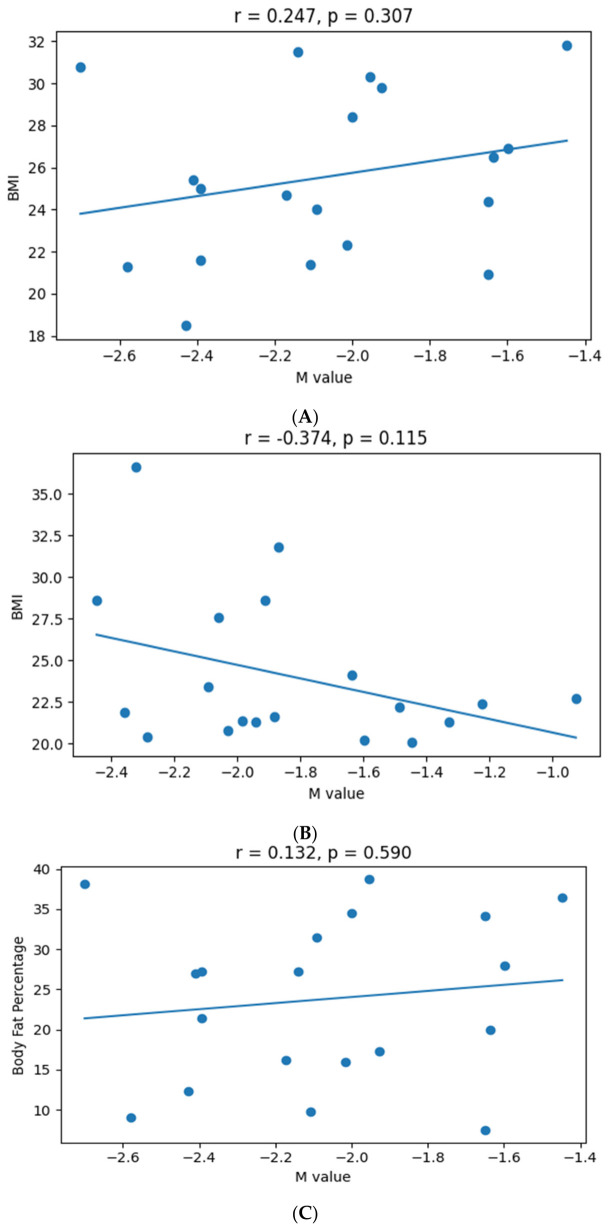

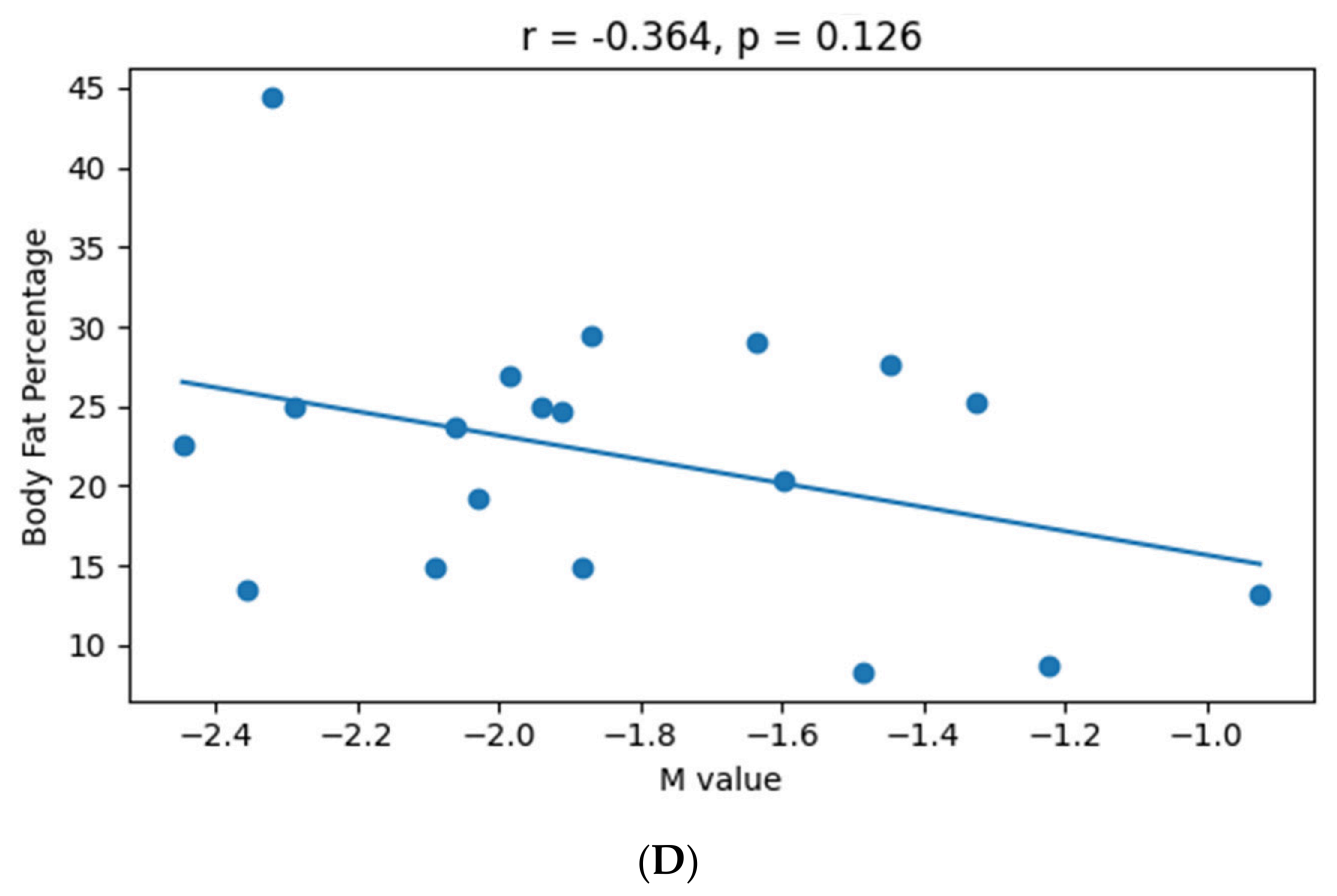
(**A**) Correlation analysis of M-value vs. BMI in the sedentary, light and moderate PA level groups. (**B**) Correlation analysis of M-value vs. BMI in the vigorously active PA level group. (**C**) Correlation analysis of M-value vs. BFP in the sedentary, light and moderate PA level groups. (**D**) Correlation analysis of M-value vs. BFP in the vigorously active PA level group.

Pyrosequencing was completed for three CpG sites close to the promoter TSS in the *SREBF1* gene in all 56 samples and digital methylation percentage (MP) results were generated for 38 samples. In total, 18 samples were excluded due to quality fails. M-values were derived from beta (B) values using the formula M = log_2_(B/(1 − B)). Figure 3 shows a comparison between the SLPA and MPA groups vs. VPA group, indicating *SREBF1* is hypermethylated in the VPA group (*p* = 0.0704). A correlation analysis did not identify any significant associations between M values and BMI or BFP in PA groups (Figure 4A–D). No significant differences were found between M-value data sets for males and females (*p* = 0.8682), or for younger adults and older adults (*p* = 0.0912).

## 3. Discussion

Obesity is a multifactorial condition which has become more widespread in recent years, attributed in part to increasingly sedentary lifestyles [18,31]. Our cross-sectional pilot study aimed to improve our understanding of the genetic and epigenetic influence of genes associated with obesity by investigating relationships between PA and BC. We recruited a healthy adult cohort, eliminating confounding factors such as smoking and underlying disease or long-term medication. We completed genotyping for the SNPs *FTO* rs9939609 and *MC4R* rs17782313, and investigated possible associations with PA and BC. We conducted methylation analyses of three CpG sites in the *SREBF1* promoter region using pyrosequencing, and as a novel investigation, analysed methylation differentials based on PA levels to understand the effect that PA may be having on the regulation of the *SREBF1* gene and its role in BC.

The role of the *FTO* gene in obesity development is well established and was the first gene identified through GWAS as contributing to polygenic obesity [13]. Expressed at high levels in the hypothalamus, it functions as an Alk-B-like enzyme that mediates demethylation of N6-methyladenosine in messenger RNA, and influences gene expression involved with energy homeostasis [32]. *FTO* shows functional interaction with other obesity-associated genes including insulin-like growth factor (*IGF-1*), known to influence carbohydrate and lipid metabolism, and Iroquois homeobox 3 (*IRX3*), which has a role in adipose browning [15,16]. Presence of the risk allele A in *FTO* rs9939609 has been found to be more likely to predict or contribute to increased BFP than the wildtype TT. The homozygous *FTO* rs9939609 A risk variant has also been linked with reduced satiety with higher levels of circulating post-prandial ghrelin and a preference for energy-dense foods [33]. Associations have been made between regular participation in PA and healthier eating behaviours, whereas lower PA levels and a sedentary lifestyle were associated with unhealthy eating behaviours including compulsive or emotional eating and overconsumption contributing to weight gain [34].

Our study identified a strong association between *FTO* risk variants and a likelihood of increased BMI in the SLPA group, and a likelihood of increased BFP in the SLPA and MPA groups through the odds ratio analysis. There was also a significant negative correlation between the *FTO* rs9939609 A risk allele and BFP in our cohort’s VPA group (*p* = 0.0387). This is consistent with a recent study that also found an association between regular PA and favourable BC in carriers of the risk allele [12] and contrasts with findings of previous studies establishing a significant positive association between *FTO* rs9939609 SNPs and BFP [15,16]. As there is a higher proportion of *FTO* rs9939609 A risk alleles in our cohort VPA group, we propose that the vigorous level of PA in this group helps to mitigate these risks, demonstrating that weight gain is not inevitable for carriers of this SNP. This assertion has been made for other *FTO* SNPs such as rs1121980 and supports the idea that a physically active lifestyle can help to combat genetic susceptibility to obesity [35], and that becoming overweight is not inevitable in those with a predisposition to weight gain [18].

*MC4R* is a member of the G-protein-coupled receptor family and is a key component in the leptin–melanocortin pathway. It is expressed at high levels in the brain and its primary functions involve regulating food intake, satiety signalling, energy homeostasis, and body weight [9]. The influence of the *MC4R* gene polymorphisms on obesity is well studied, with the rs17782313 C risk allele linked to loss of function and increased BMI. Several cohort studies have established a positive association between this SNP variant and increased BMI [16,36] and our study has also identified a significant positive association between the *MC4R* rs17782313 C risk allele and BMI in the VPA group (*p* = 0.0256). Our odds ratio analysis identified strong associations between *MC4R* variants and a likelihood of increased BMI in all PA level groups, and a likelihood of increased BFP in the VPA group. As BMI does not distinguish between different tissue types such as fat mass and non-fat mass or muscle mass, a higher BMI would not necessarily indicate an unhealthy body composition in an exercising population [4,12]. This highlights the importance of using multiple body composition measures to allow for more accurate interpretation of findings [4].

*SREBF1* is known to contribute to obesity through its role as a key transcriptional regulator of lipid metabolism, interacting with key lipogenic genes such as fatty acid synthase (FAS) and stearoyl-CoA desaturase (SCD1). Inhibition of *SREBF1* expression has a controlling effect on lipid synthesis [37] while hypomethylation of *SREBF1* has been linked with increased cholesterol biosynthesis and accumulation in human adipocytes [26]. Although our *SREBF1* CpG site methylation findings did not indicate any significant differentials between PA groups, we observed a higher rate of methylation across all three CpG sites for the VPA group. Age and sex can be confounding factors in DNAm analysis [38,39], although we found no significant differences in the results between males and females, or between younger and older adults. As *SREBF1* is known to interact with several lipogenic genes such as *FAS* and *SCD1*, further exploration of gene expression in these pathways in a greater number of participants would be beneficial to improve our understanding of these gene–gene interactions [40].

PA is known to induce differing physiological responses in males and females, and the effect of PA on DNAm by sex is established through global methylation and certain genes linked to sex. Biological sex differences in DNAm are not well established with all the *FTO*, *MC4R* and *SREBF1* genes linked to BMI, BFP and cholesterol metabolism, respectively [38,41]. DNAm profiles can vary with age, with DNAm changes induced by PA thought to be more pronounced in older age groups [39].

The strengths of our study include the tightly controlled criteria for participant inclusion in the cohort, and the consistency of participant PA habits over the previous six months. A review of PA questionnaire responses with participants in the clinic helped to reduce inaccuracies associated with self-reporting of PA. The exclusion of participants with a history of smoking or vaping, recent or long-term medication, underlying health conditions, metabolic disorders, and pregnancy or lactation allowed for control of confounding factors known to influence methylation [42,43]. These restrictions limited participation numbers and the study was somewhat underpowered, so expanding the cohort for future investigations would help to explore these initial findings further. More in-depth study of the epigenetic influence of PA on the *SREBF1* gene and regulation of gene expression in this pathway will help to further develop our understanding of the interaction of PA with genes associated with obesity.

## 4. Materials and Methods

*a.*  *Optimal sample size planning*

Recommended cohort size was determined using a priori analysis. G*Power 3.1.9.7 software [28] suggested a total sample size of 82 participants based on a two-tailed test with effect size of 0.3 and α error probability of 0.05 to achieve a required power of 0.80.

*b.*  *Ethics and study design*

This is a cross-sectional study and all procedures were approved by the University of Derby ethics committee, ref number ETH-2324-3589. All study participants provided written and informed consent in accordance with the Declaration of Helsinki.

*c.*  *Participant recruitment*

Recruitment took place between August 2024 and November 2025. Participants were between 18 and 65 years of age, males and females, non-smokers or -vapers, with no underlying health conditions or long-term prescription medication, and were not pregnant or lactating. All PA and BC levels were considered. The cohort was recruited from the local university staff and student population using email and poster campaigns. Participants completed an online health questionnaire to check that the eligibility criteria for the study had been met, and an IPAQ [30] was used for PA level classification. PA level descriptions were based on UK Government Chief Medical Officer weekly recommended guidelines [5]. Once the questionnaires had been screened for suitability, the participants were invited to attend a clinic appointment to complete their participation in the study.

*d.*  *Clinic data collection*

Upon arrival at the clinic at the University of Derby, the researcher briefly discussed the questionnaire responses with the participant to check if there were any changes to health or PA habits since completion. The participant was asked about the consistency of their PA habits and any injuries affecting their PA over the last six months to confirm the PA level classification. Classification of the self-reported PA levels were reviewed with a second researcher for consistency after the clinic appointment, and this information was used to clarify any classifications that were unclear from the IPAQ [30]. Specifically, 150 min of MPA or 75 min of VPA or a combination of both was considered as meeting the government target and classed as moderate; below this level was classed as light, and in excess of this level was classed as vigorous; less than 30 min activity a day was classed as sedentary.

Body composition measurements were taken using a stadiometer for height, and weighing scales were used to calculate BMI. BFP was estimated using bioelectrical impedance scales (Tanita DC-240 Body Composition Analyser, Amsterdam, The Netherlands). BMI ranges were classified according to NICE guidelines as healthy, overweight and obese [2] (Table 5). BFP ranges were classified into athlete, fitness, average, and obesity, using different ranges for males and females, according to guidelines from the ACSM [29] (Table 6). A venous blood sample of approximately 5 mL was taken from the upper arm using a heparin-coated vacutainer and transported to the laboratory within the University of Derby premises for processing.

*e.*  *Blood sample processing and DNA extraction*

Whole blood samples were buffered with an equal volume of phosphate-buffered saline with foetal bovine serum at 2% (PBS/FBS 2%) in a 15 mL falcon tube and centrifuged at 3000 RPM for 15 min at room temperature (Eppendorf Centrifuge 5804R, Hamburg, Germany). The plasma layer was removed and the leukocyte-rich buffy coat layer used for DNA extraction. DNA was extracted using a QIAamp DNA micro kit (Qiagen, cat 56304, Velno, The Netherlands) in accordance with the manufacturer’s instructions, quantified using a nanodrop (Thermo Fisher Scientific Nanodrop 2000, Waltham, MA, USA, software version 1.6.198), and maintained in −20 °C freezer storage.

*f.*  *Genotyping*

Genotyping was completed using TaqMan^®^ SNP assays (ThermoFisher Scientific, cat 4351379) for two genes of interest (Table 7). A total of 10 ng of purified genomic DNA in nuclease-free H_2_O was added to 12.5 µL TaqMan^®^ Genotyping Master Mix (ThermoFisher cat no 4371353, Waltham, MA, USA) and a 1.25 µL SNP assay in a 96-well plate was performed in duplicate according to the manufacturer’s instructions. The plate was analysed using qPCR (Applied Biosystems StepOnePlus Real-Time PCR System qPCR, Waltham, MA, USA). A genotyping programme was used for end-point analysis to identify the target alleles (StepOne^TM^ Software v2.2.2, Waltham, MA, USA) and results were interpreted using TaqMan^®^ Genotyper Software (version 1.7.1, ThermoFisher, Waltham, MA, USA).

*g.*  *Pyrosequencing*

Pyrosequencing was performed using the PyroMark^®^ Q48 Autoprep Instrument (cat no 9002471, Qiagen, Velno, The Netherlands). Genomic DNA samples were bisulfite-converted and cleaned up using an Epitect Bisulfite kit (cat no 59104, Qiagen, Velno, The Netherlands) according to manufacturer’s instructions. Bisulfite-converted DNA was prepared using a PyroMark PCR kit (cat no 978703, Qiagen, Velno, The Netherlands) and the primer set from the CpG assay for the *SREBF1* gene region (cat no 978746, Gene Globe ID PM00178087, Hs_*SREFB1*_01_PM, Qiagen, Germany) according to the manufacturer’s instructions, with an Epitect PCR control DNA set (cat no 59695, Qiagen, Velno, The Netherlands). A 2% agarose gel was used to check for the expected amplicon fragment size of 136bp. The pyrosequencing was performed with the sequencing primer from the CpG assay detailed above, using PyroMark^®^ Q48 Advanced Reagents kit, PyroMark^®^ Q48 Magnetic Beads, and PyroMark^®^ Q48 discs (cat no 974002, 974203 and 974901, respectively, Qiagen, Velno, The Netherlands). A PyroMark^®^ Control Oligo was used to verify the correct functioning of the instrument as an internal quality check (cat no 979203, Qiagen, Velno, The Netherlands). The PyroMark^®^ Q48 software (cat no 9024325, Qiagen, Velno, The Netherlands) was programmed using sequencing details provided with the assay (Table 8). Each sample was sequenced in duplicate using two identical subsequent runs.

*h.*  *Statistical analysis*

Statistical analysis was performed using GraphPad Prism (version 11.0.2(92), Boston MA, USA) and Python (version 3.1).

BMI and BFP data sets were checked for normal Gaussian distribution using a D’Agostino & Pearson test and a histogram was generated to check for a normal bell curve. Participants were grouped according to their PA level and the genotypes *FTO* rs9939609 and *MC4R* rs17782313 allele variants. Risk allele variants were grouped for the purpose of comparison with the healthy version of the gene. Odds ratios were calculated by grouping participants into less active (sedentary/light) and more active (moderate/vigorous) PA groups for binary analysis. The two-sided Fisher’s exact test was used to calculate *p* values. Effect sizes were analysed using odds ratios (OR) with 95% confidence intervals (CI) computed using the Baptista–Pike method. For correlation analysis, participants were grouped by their PA level as sedentary/light, moderate, and vigorous. This was completed using the two-tailed non-parametric Spearman test to determine possible genotype association with BMI and BFP parameters. Results were considered statistically significant if *p* ≤ 0.05. A multiple logistic regression analysis was performed to check for confounding factors including age and sex.

M-values were derived from beta (B) values using the formula M = log_2_(B/(1 − B)), calculated in Microsoft Excel (Version 16.0 Microsoft excel for MS365). MP above 50% produced a positive value, and MP below 50% produced a negative value, providing data more suitable for statistical analysis.

M-value data sets were checked for confounding factors of age and sex using a non-parametric unpaired *t*-test and a Mann–Whitney rank test. Age groups were categorised as a younger adult age group (18–45 years) and an older adult age group (46–65 years), based on European Medicines Agency (EMA) biological guidelines [44].

Pyrosequencing M-values were analysed using Python for each group and a bar plot. The analysis was carried out using libraries such as pandas for data handling, NumPy Version 2.4.6 for numerical computation, and Matplotlib for visualisation.

## Figures and Tables

**Table 3:** (**A**) Odds ratio analysis for PA levels and BC for *FTO* rs9939609. Total *N* = 55; *n* values in table indicate number of participants in the health risk BC group by PA level. Strong associations with the risk variant were identified for BMI in the SLPA group and for BFP in the SLPA and MPA group. (**B**) Odds ratio analysis for PA levels and BC for *MC4R* rs17782313. Total *N* = 56; *n* values in table indicate number of participants in the health risk BC group by PA level. NC = not calculated due to 0 participants in healthy BFP SLPA group for risk variant. Strong associations with the risk variant were identified for BMI in all PA groups and BFP in the VPA group.

(**A**)				
**BC Group**	**PA Level**	**Odds Ratio**	**95% CI**	***p* Value**
BMI overweight/obese	All PA *n* = 27	0.7792	0.2339 to 2.723	0.6947
	SLPA *n* = 10	2.333	0.2509 to 19.50	0.4805
	MPA *n* = 6	0.5000	0.02827 to 6.096	0.6210
	VPA *n* = 11	0.8438	0.1497 to 5.473	0.8655
BFP average/obesity	All PA *n* = 34	1.016	0.3020 to 3.509	0.9810
	SLPA *n* = 12	2.000	0.08546 to 42.12	0.6488
	MPA *n* = 5	2.000	0.1640 to 35.37	0.6210
	VPA *n* = 17	0.8485	0.1333 to 4.785	0.8691
(**B**)				
**BC Group**	**PA Level**	**Odds Ratio**	**95% CI**	***p* Value**
BMI overweight/obese	All PA *n* = 27	1.762	0.6406 to 5.170	0.2951
	SLPA *n* = 10	3.000	0.3142 to 45.71	0.3932
	MPA *n* = 6	1.762	0.6406 to 5.170	0.2951
	VPA *n* = 11	1.944	0.3930 to 9.597	0.3895
BFP average/obesity	All PA *n* = 35	1.535	0.5236 to 4.254	0.4452
	SLPA *n* = 12	NC	NC	NC
	MPA *n* = 5	0.3000	0.01769 to 3.867	0.3869
	VPA *n* = 18	2.667	0.6092 to 11.09	0.2198

**Table 4:** (**A**) Associations between PA levels and anthropometrics, with interaction effects by *FTO* rs9939609. *n* = 55. Completed using Spearman’s correlation analysis. (**B**) Associations between PA levels and anthropometrics, with interaction effects by *MC4R* rs17782313. *n* = 56. Completed using Spearman’s correlation analysis (* Indicates *p* < 0.05).

(**A**)				
**BC**	**PA Level**	**Spearman’s r**	**95% CI**	***p* Value**
	SLPA	−0.1048	−0.6130 to 0.4646	0.7193
BMI	MPA	0.1168	−0.5343 to 0.6809	0.7719
	VPA	−0.06820	−0.4273 to 0.3095	0.7203
	SLPA	−0.04193	−0.5719 to 0.5128	0.8874
BFP	MPA	0.1561	−0.5051 to 0.7018	0.6595
	VPA	−0.3793	−0.6570 to −0.01089	0.0387 *
(**B**)				
**BC**	**PA Level**	**Spearman’s r**	**95% CI**	***p* Value**
	SLPA	−0.05121	−0.6193 to 0.5521	0.9333
BMI	MPA	−0.1140		0.8333
	VPA	0.4070	0.04368 to 0.6753	0.0256 *
	SLPA	0.4297	−0.1478 to 0.7887	0.1419
BFP	MPA	−0.2264	−0.7370 to 0.4486	0.5273
	VPA	0.3131	−0.05733 to 0.6077	0.0864

**Table 5:** BMI ranges according to NICE guidelines [2]. BMI is calculated as mass (kg)/height(m)^2^.

BMI Group	BMI Range
Healthy	18.5–25
Overweight	25.1–30
Obese	>30.1

**Table 6:** BFP ranges according to ACSM classification [29]. Classifications are based on separate ranges for males and females to reflect biological differences in body composition.

BFP Classification	Male	Female
Athlete	6–13%	14–20%
Fitness	14–17%	21–24%
Average	18–24%	25–31%
Obesity	>25%	>32%

**Table 7:** Target gene SNP assays used with alleles of interest identified. The VIC/FAM labels were used for software identification, and ROX dye used as the passive reference. Assays obtained from ThermoFisher Scientific (ThermoFisher, Waltham, MA, USA, cat #435179).

Gene	SNP Analysed	Assay ID	VIC Allele	FAM Allele
*FTO*	rs9939609	C_30090620_10	A	T
*MC4R*	rs17782313	C_32667060_10	C	T

**Table 8:** Pyrosequencing Pyromark Q48 software (cat no 9024325, Qiagen, Velno, The Netherlands). Details of assay used to quantify methylation levels of CpG sites in *SREBF1* gene region, obtained from the manufacturer’s website (Qiagen, Velno, The Netherlands).

Feature	Details
Sequence to analyse	ACGGCTGAGCTCCGGGCACCGA
Sequence after bisulfite treatment	AYGGTTGAGTTTYGGGTATYGA
Nucleotide dispensation order	TATCGTCTAGAGATTCGTAGTCG
Amplicon length	136 bp
Sequenced strand	Antisense
CpG sites included	3
Chromosomal location GRCh38	Ch17, bp178406XX
Assay description	Hs_*SREBF1*_01_PM PyroMark CpG assay
Assay details	Cat # 978746, PM00178087

## Data Availability

Data supporting the results presented in the paper is available upon request.

## Abbreviations

The following abbreviations are used in this manuscript:

ACSM	American College of Sports Medicine
B-value	Beta values (average methylation percentage)
BC	Body composition
BFP	Body fat percentage
BMI	Body mass index
CI	Confidence intervals
CpG	Cytosine–phosphate–guanine
CVD	Cardiovascular disease
DNA	Deoxyribonucleic acid
EMA	European Medicines Agency
FAS	Fatty acid synthase
FBS	Foetal bovine serum
FTO	Fat mass and obesity-associated gene
GWAS	Genome-wide association studies
IGF-1	Insulin-like growth factor
IPAQ	International Physical Activity Questionnaire
IRX3	Iroquois homeobox 3
M-value	Methylation value
MP	Methylation percentage
MC4R	Melanocortin-4 receptor gene
MPA	Moderate physical activity
NICE	National Institute for Health and Care Excellence
OR	Odds ratios
PA	Physical activity
PBS	Phosphate-buffered solution
PCR	Polymerase chain reaction
qPCR	Quantitative polymerase chain reaction
SCD1	Stearoyl-CoA desaturase
SDS	Standard deviation
SLPA	Sedentary and light physical activity
SNP	Single-nucleotide polymorphism
SREBF1	Sterol Regulatory Element Binding Transcription Factor 1
T2D	Type II diabetes
TSS	Transcription start site
VPA	Vigorous physical activity
WHO	World Health Organisation
